# Impact of the halitosis on QoL in overweight and obese patients: Based on six years of experience in two centers in sulaimani governorate, Kurdistan Region/Iraq, and case series study

**DOI:** 10.1016/j.amsu.2019.05.008

**Published:** 2019-05-30

**Authors:** Hiwa Omer Ahmed, Sherko Saeed F. Zmnako, Zanyar Mustafa Amin, Rajan Fuad Ezzat, Aram Kakarash, Shahen H. Omer, Hawbash Othman, Bawan Sherif

**Affiliations:** a Professor & Senior Lecturer in College of Medicine, University of Sulaimani, Iraq; b Otolaryngologist Senior Lecturer in College of Medicine, University of Sulaimani, Iraq; c Maxillofacial Surgeon, Senior Lecturer in College of Medicine, University of Sulaimani, Iraq; d General Surgeon, Sulaimani Teaching Hospital, Iraq; e Kurdistan Board Trainee in Maxillofacial surgery, Iraq; f Kurdistan Board Trainee in general surgery, Iraq

**Keywords:** Overweight, Obesity, Halitosis, Emotional aspects, Social aspects

## Abstract

**Background/objectives:**

The patients who are overweight and obese, are under stress of excess body weight, embarrassed, one may imagine the impact of halitosis on this group of patients, this is an attempt to evaluate this extra impact, and which aspect of quality of life will be affect in the overweight and obese.

**Subjects/methods:**

A prospective case series study including 885 overweight or obese patients, they were consulting for advice, diet and or drugs and various bariatric operations. A group of normal weight patients with halitosis, matched in age group and gender were enrolled as a control group for comparison. Patients who have either oral causes of the condition or pseudo halitosis or halite-phobia or were using drugs like phenytoin, cyclosporine or **calcium** channel blockers, isosorbide di-nitrate, Chloral hydrate, Nitrites and Nitrates, Dimethyl sulphoxide, Disulphiram, cytotoxic agents, Phenothiazine were excluded.

**Interventions/methods:**

The work conducted over a period of 6 years from February 1st, 2012 to March 1st, 2018. Prospective evaluation of the type and etiology of halitosis was done by using organoleptic measurement, which is not a slandered but evaluated by a group of academic colleagues. The patients were advised to avoid eating odiferous foods for 48 hours before the assessment and both the patient and the examiner should refrain from drinking coffee, tea or juice, smoking and using scented cosmetics before the assessment.

**Results:**

Halitosis in the overweight and obese patients magnifies the negative aspects of quality of life: avoidance, narrow social circle (P Value = 0.3415, 95% confidence interval = 11.43924–29.67085), avoidance of sex by partner (P Value = 0.0143, 95% confidence interval = 04.11537–17.08480), low self-esteem (P Value = 0.0100, 95% confidence interval = 10.66794–28.44776), teasing by others and negative thoughts (P Value = 0.4013, 95% confidence interval = 11.43924–29.67085). While obesity was not a cause of avoidance of to be kissed by partner in obese patients, but was a direct cause for this avoidance in obese patients with halitosis (P Value = 0.0143, 95% confidence interval = 04.11537–17.08480). Halitosis in normal weight patients affects the quality of life **remarkably** but not to the extent of halitosis in overweight and obese patients, especially social life and self-esteem which will suffer most.

**Conclusions:**

The quality of life of overweight and obese, especially emotional and social aspects was significantly disturbed by halitosis more than normal weight subjects with halitosis.

## Highlights

• Halitosis is a common problem in general population.
• There is a relation of overweight and obesity with some oral conditions causing halitosis.
• The patients who are overweight and obese, are under stress of excess body weight.
• One may imagine the impact of halitosis on patients who are overweight and obese.
• Halitosis in the overweight and obese patients magnifies the negative aspects of quality of life.

## 1 Introduction

“Halitosis” is a medical term originated by a Latin word (halitus; breathed air)) and a Greek suffix (-osis; pathologic alteration), and is utilized to depict any unsavory awful or upsetting odor rising out of the mouth that is recognized by others [1,2].

Halitosis is very common, 25(3) to 50% of the general population have halitosis. In the Swedish only present in around 2% of the population, in China the incidence is above 27% [4,5]. Both genders suffer the same [3].

Although halitosis has multifactorial origins, the source of 80–90% cases is oral cavity [5,6], results from tongue coating, periodontal disease, peri-implant disease, deep carious lesions, exposed necrotic tooth pulps, pericoronitis, mucosal ulcerations, healing (mucosal) wounds, impacted food or debris, imperfect dental restorations, unclean dentures and factors causing decreased salivary flow rate [3,4,7,8]. An association between overweight and obesity and periodontitis was mentioned in the literature, it may be due to an increased local inflammatory response as well as possibly an altered periodontal microflora (9.10, 11, 12).

In the remaining 9% of patients, the source of halitosis is non-oral reasons such as urinary system, hepatic, pancreatic, trimethylaminuria, and upper and lower respiratory tract infection and gastrointestinal system. In 1% of patients, the cause of the halitosis is diets or drugs [3,5]. Gastroesophageal reflux disease (GERD) [3], [13], [14], [15], [16], [17], [18], [19] and *Helicobacter pylori* infection are the leader causes of gastrointestinal halitosis [1,3,15]. Gastroesophageal reflux disease (GERD) has predilection for females, and affects up to 20(16, 20) to 40% of the population. Obesity and GERD are clearly related, symptoms increase in severity when people gain weight [18,21]. As social relations are one of the domains in the quality of life construct, halitosis needs to be considered as a factor of negative interference [22].Halitosis has a negative effect on a person's social life. The person who has halitosis may not be aware of this situation because this person may have developed tolerance or olfactory disturbance [1], albeit identified by his/her partner, family member, or friends. It has a large psychological, social and economic impact [3,17], and these situations affect individual's relation with other people [5], affects their social communication and life [2,3], may avoid socializing [1].

The patients who are overweight and obese, are under stress of excess body weight, embarrassed [23], one may imagine the impact of halitosis on this group of patients, this is an attempt to evaluate this extra impact, and which aspect of quality of life (QoL) will be affected in the overweight and obese, the patients in this case series unified by their extra body weight and halitosis.

## 2 Patients, materials and methods

A prospective case series study including 885 overweight or obese patients, they were consulting for advice, diet and or drugs and various bariatric operations. This was follow the SCARE guidelines for the reporting [24] and conducted over a period of 6 years from February 1st, 2012 to March 1st, 2018.

Each patient was evaluated clinically with body mass index (BMI), Excess body weight (EBW) calculated from ideal body weight (IBW), measured on first and each consequent visits. All patients underwent complete evaluation before the operation (including endoscopy and abdominal ultrasonography). Additional investigations were performed according to the risk profile of each individual patient.

Blood and biochemical workup were done for each patient. For the collection of the required information, each patient was interviewed face-to-face, by three trainees (from Kurdistan Board for Medical Specialties/surgery) who were working in the following centers: Hatwan private hospital, and Sulaimani Teaching Hospital. An originally –designed questionnaire was filled out in English language, translated to Kurdish language by the interviewer, which was not slandered and evaluated by a group of colleagues, before application in the interviews. It was composed of demographic, medical and biological data. An informed consent was obtained and a consent form was signed by each patient. The research was confirmed by the Ethics Committee of the University of Sulaimani, College of Medicine number 2 in 2013, and registered in research registry with researchregistry UIN 4430.

Prospective evaluation of the type and etiology of halitosis was done by using organoleptic measurement, which is not a slandered but evaluated by a group of academic colleagues. The patients were advised to avoid eating odiferous foods for 48 h before the assessment and both the patient and the examiner should refrain from drinking coffee, tea or juice, smoking and using scented cosmetics before the assessment [6].

During organoleptic measurement: each patient takes a deep breathe by inspiring air by nostrils and holding awhile, then expiring via a pipette, while the examiners sniff the odor at a distance of 20 cm and the severity of odor is classified into various scales, such as a 0- to 5-point scale(3). While for evaluation of extra-oral halitosis, patients were sked to slowly exhale air out of the nose, at a distance of approximately 20 cm from the nose of the examiner.

Dentist and Maxillofacial surgeons were examining the patients to exclude oral factors that contribute to halitosis like surgical, and pathologic factors such as exposed tooth pulps and non-vital tooth with fistula draining into the mouth, oral cavity pathologies, oral cancer and ulcerations, extractions/healing wounds or prosthetics or dentition factors such as orthodontic fixed appliances, keeping at night or not regularly cleaning dentures, restorative crowns which are not well adapted, non-cleaning the bridge body, and interdental food impaction, poor oral hygiene, food debris and dental bacterial plaque accumulated on the teeth and tongue, and caries and periodontal diseases like gingivitis, periodontitis, and salivary flow reduction or xerostomia.

ENT team was examining the patients to exclude potential nasal and pharyngeal causes of halitosis.

Psychological checkup aimed in exclusion of halitophobia, psychosomatic halitosis to enroll those with genuine halitosis.

Patients who have either oral causes of the condition or pseudo halitosis or halitophobia or were using drugs like phenytoin, cyclosporine or calcium channel blockers [5], isosorbide di-nitrate, Chloral hydrate, Nitrites and Nitrates, Dimethyl sulphoxide, Disulphiram, cytotoxic agents, Phenothiazines were excluded.

All the (885) patients were examined for halitosis during the 6 year study timeframe, a group of normal weight patients with halitosis, matched in age group and gender were enrolled as a control group for comparison, they also underwent examination by all the teams, and were arranged in 2 groups, similarly a subgroup group of overweight and obese patients without halitosis, matched in age group, gender and BMI were enrolled as subgroup A2 group for comparison with group A1, they also underwent examination by all the teams,

Group A, the study group, subdivided into two subgroups:

A1: 30 overweight or obese patients with halitosis

A2: 30 overweight or obese patients without halitosis

Group B, control group 30 normal weight patients with halitosis

All the collected data were collected, organized and analyzed by Statistical Package for the Social Sciences (SPSS); version 21.

## 3 Results

From total of 885 overweight and obese patients 63 patients have halitosis, causes of the condition are shown in Table 1.

**Table 1:** Causes of halitosis in overweight and obese patients.

Causes of halitosis in group A			No = 63 & frequency	Female	male
Oral Cavity	Deeply carious tooth		13 20.63%	4	9
	Tongue coating		2 03.17%	1	1
	Periodontal disease		5 07.93%	1	4
	Inter-dental food impaction		3 04.76%	1	2
	Impacted food or debris		4 06.34%	2	2
	Exposed necrotic tooth pulps		6 09.52%	4	2
Non-oral causes	Respiratory		0 0.0%	0	0
	Hepatic		0 0.0%	0	0
	Renal		0 0.0%	0	0
	Gastrointestinal	GERD	15 23.80%	3	12
		H pylori infection	10 15.87%	4	6
Lifestyle	Rapid eating and inadequate chewing		5 07.93%	1	4

Thirty three patients were excluded because either they have oral causes, pseudo halitosis or halitophobia or were using drugs like phenytoin, cyclosporine or calcium channel blockers [5], isosorbide di-nitrate [19] which may increase the risk of bad odor.

No any patients with halitosis have had respiratory, renal or hepatic halitosis.

In five patients of the reaming 30, they had no classified cause for their halitosis, Apart from habits of rapid eating and inadequate chewing of foods.

From a total of 63 patients, 33 patients have oral cavity causes, no any patient have systemic s, and 5 patients of the remaining 30 patients, had no any classified physical causes apart from bad lifestyle habits of obese patients like rapid eating and not chewing properly. While 25 patients have gastrointestinal causes, the majority had GERD and 10 of them have only *Helicobacter pylori* infection. Sixty three (07.11%) of the patients with genuine halitosis, nearly half of them (n = 30, 3.38%) have extra oral causes for their condition, 25 (02.82%) patients with gastrointestinal causes; 15 patients (01.69%) were with GERD, 10 (01.13%) with Helicobacter Pylori, while 5 patients (00.56%) have wrong lifestyles, i.e. rapid eating and inadequate chewing (Table 1).

In conclusion of the examination of the obese patients with abnormal odor from mouth and nose, thirty two patients were excluded as shown in Table 2.

**Table 2:** Patients exclude with abnormal odor from noise and mouth.

Variables	Finding	Female	Male
Drugs	calcium channel blockers	8	3
	isosorbide di-nitrate	0	3
Psychological	halite-phobia,	5	2
	pseudo-halitosis	4	1
Others	Respiratory causes	0	0
	Renal and hepatic causes	0	0
	Otolaryngology causes	2	3

The quality of life of the overweight and obese patients, in group A compared to patients in group B, halitosis decreases the social contact, like avoidance and narrowing of social circle, increases teasing by others and dropping of the self-esteem and increases negative thoughts, to the extent most of their partners were avoid kissing them, Table 4.

**Table 3:** Comparing variables in overweight and obese patients with halitosis (group A_1_) to overweight and obese patients without halitosis (group A_2_) and normal weight patients with halitosis (group B).

Variables	Group A_1_	Group A_2_	Group B
Patients	30	30	30
Age groups	25 ± 7	25 ± 8	26 ± 7
Gender ratio M/F	1/3	1/3	0.37/1
GERD	15	0	17
H pylori infection	10	0	11
No cause found	5	0	0

**Table 4:** Comparing Aspects of QoL of overweight and obese patients with halitosis to overweight and obese patients without halitosis.

Aspects of QoL	Sub group A_1_	Sub group A_2_	P value	95% Confidence intervals
Avoidance	21 70.00%	13 43.33%	0.4627	09.90313–27.21865
Narrow social circle	22 73.33%	16 53.33%	0.3415	11.43924 -29.67085
Avoid Kissing by partner	12 40.00%	0 00.00%	0.0023	02.20189 -13.05947
Avoid sex by partner	16 53.33%	2 06.66%	0.0143	04.11537 -17.08480
Low self-esteem	25 83.33%	12 40.00%	0.0100	10.66794 -28.44776
Teasing by others	30 100.00%	20 66.66%	0.4320	16.17868 -36.90493
Negative thoughts	21 70.00%	17 56.66%	0.4013	11.43924 -29.67085
Feeling Sexually attractive	4 13.33%	20 66.66%	0.0030	06.20058 -20.96159

Obese patients with halitosis have the lowest self-esteem, all of them were teased by others, near half (n = 12, 1.35%) of them were abandoned for sex by their partners (P Value = 0.0143, 95% confidence interval = 04.11537–17.08480), while there was no obese patients without halitosis, abandoned for sex by their partners (P Value = 0.0143, 95% confidence interval = 04.11537–17.08480).

While there was no obese patients without halitosis, abandoned for sex by their partners (P Value = 0.0143, 95% confidence interval = 04.11537–17.08480), two third of the patients (n = 20, 66.66%) in subgroup A2 (obese), were feeling sexually attractive, this declined to (n = 4, 13.33) of the patients in subgroup A1, who were obese and have halitosis, Table 4.

Another comparison was done to normal weight patients with halitosis to show the effect of halitosis, Table 5, shows that halitosis in normal weight patients affects the QoL remarkably but not to the extent of halitosis in overweight and obese patients, especially social life and self-esteem which will suffer most. While patients with halitosis in overweight and obese, same as normal weight patients with hilatosis were suffering of negative quality of life; as avoided to be kissed by the partner, were avoidance, have narrow social circle and low self-esteem (20%, 70%, 73%, 75%) of the obese patients with halitosis ere versus (10%, 56%, 53%, 66%) in patients having halitosis, but have normal weight (group B) respectively, Table 5.

**Table 5:** Comparing Aspects of QoL of overweight and obese patients with halitosis (group A_1_) to normal weight patients with halitosis (group B).

Aspects of QoL	Group A_1_	Group B	P Value	95% Confidence intervals
Teasing by others	30 100.00%	8 26.66%	0.03866	07.65393 -03.48962
Low self-esteem	25 75.00%	20 66.66%	0.53766	14.58003 -34.51129
Narrow social circle	22 73.33%	16 53.33%	0.51190	11.43924 -29.67085
Avoidance	21 70.00%	17 56.66%	0.42870	11.43924 -29.67085
Negative thoughts	21 70.00%	11 36.66%	0.02622	09.14538 -25.98300
Avoid Kissing by a partner	12 40.00%	8 26.66%	0.43520	04.79539 -18.39036
Avoid sex by a partner	6 20.00	3 10.00%	0.70935	01.62349 -11.66833
Feeling Sexually attractive	4 13.33%	18 60.00%	0.01400	05.49116 -19.68204

One could see the effect of halitosis clearly, as both group B (normal weight) and subgroup A1 (obese with halitosis), have same problems but more in obese with halitosis, for example avoidance be kissed by a partner, avoidance, narrow social circle, low self-esteem, were frequent in obese patients with halitosis (subgroupA1); (40%, 70%,73%,75%) versus (26%, 56%, 53%, 66%) in patients having halitosis, with normal weight (group B) respectively. Meanwhile all the obese patients having halitosis (100%) were teased by others versus (26%) of normal weight patients with halitosis. Only minority (13%) of the patients in subgroup A1 felt sexually attractive versus (60%0 of the normal weight patients with halitosis.

## 4 Discussion

A relation of overweight and obesity with some oral conditions causing halitosis was mentioned in the literature like periodontitis and a dysbiotic inflammatory disease [10,11,23,25], besides this a good number of overweight and obese patients are eating rapidly, not chewing adequately [12,26], have dry mouth and coating of tongue [12,27], food particles may remain between teeth and cause unpleasant odor from the mouth [3,4,7,8] with intensity beyond a socially acceptable level [27,28].

Thorough dental, maxillofacial, Otolaryngologic, respiratory, abdominal examination were carried out by the dentist, maxillofacial team, internist and surgeons, beside psychological assessment for all those with halitosis, to classify the type of the halitosis and to exclude halite-phobia, pseudo-halitosis [3,19,29] to enroll only those with genuine halitosis. To exclude those using drugs like phenytoin, cyclosporine or calcium channel blockers [5], isosorbide dinitrate [19], Chloral hydrate, Nitrites and Nitrates, Dimethyl sulphoxide, Disulphiram, cytotoxic agents, Phenothiazines [29] which may increase the risk of halitosis. A number of patients (n = 32) were excluded (eleven and three patients were using calcium channel blockers and isosorbide dinitrate respectively, and 7 and 5 patients having Halito-phobia, pseudo-halitosis in this turn and 5 patients have Otolaryngologic causes (postnasal drip, chronic sinusitis with nasal septal deviation) were excluded, see Table 2.

Sixty three (07.11%) of the patients with genuine halitosis, nearly half of them (n = 30, 3.38%) have extra oral causes for their condition (Table 1), this is not in the line with literature as 90% of halitosis are caused by oral conditions [5,[9], [10], [11], [12]], this discrepancy may be explained by the facts that, there is more gastrointestinal causes in overweight and obese patients; The pH of stomach acid is much lower than normal [10], acidic contents of the stomach can reach the nasopharynx and cause irritation of its walls, resulting in postnasal drip [30]. there is a higher incidence of GERD in obese patients, impaired lower esophageal sphincter function in subjects with GERD allows intestinal gas and stomach contents to reflux into the esophagus [18,20,21,30,31], direct acid-peptic injury to susceptible supra-esophageal tissue may produces halitosis. **Ten (01.13%) of the obese patients** with halitosis (Table 1), were infected by H pylori, this infection by itself produces halitosis [24,32].

Five patients with genuine halitosis, do not have any physical causes, but they have wrong lifestyle in eating, as they were eating rapidly and not chewing the bite adequately. [12,26,27], or it may be due to the controversial link of obesity as it to halitosis [33].

To know the effect of halitosis on the Qol of obese patients, these patients were compared to a two groups of patients marched in age and gender in normal weight patients having halitosis(group B) and marched to age, gender and BMI for obese patients having no halitosis (subgroup A2) as shown in Table 3. The halitosis will affect most aspects of quality of life in normal weight patients [22,33,and35]], most aspects were affected in the normal weight patients (Table 4), but the affect were more when the patients are obese and have halitosis (Table 4, Table 5).

Obese patients with halitosis have the lowest self-esteem, all of them were teased by others, near half (n = 12, 1.35%) of them were abandoned for sex by their partners while there was no obese patients without halitosis, abandoned for sex by their partners, these notion were confessed also by the husbands, Table 5.

One may notice that halitosis in the obese patients magnifies the negative aspects of QoL: avoidance, narrow social circle, avoidance of sex by partner, low self-esteem, teasing by others and negative thoughts. While obesity was not a cause of avoidance of kissing by partner in obese patients, but was a direct cause for this avoidance in obese patients with halitosis. The patients with halitosis in overweight and obese, same as normal weight patients were suffering of negative quality of life; as (20%) of the obese patients with halitosis ere avoided to be kissed by the partner, while (10%) in patients having halitosis, but have normal weight (group B) respectively. The kiss is a demonstration of affection, a symbolic gesture of affirmation and bonding with another person, social relations and self-esteem [22].

Quality of life is influenced by the satisfaction and happiness felt subjectively by an individual through social relations and complexities, such as psychological state and physical health [35] Halitosis was highlighted to have a link to quality of life, is considered as a social impediment [22], to have a strong emotional impact on their quality of life [35], may cause embarrassment, depression and make relationships more difficult [[36], [37]].

People suffering from halitosis create a social barrier between themselves and their friends, relatives, partners or colleagues at work [11,17,22], Current social norms emphasize the importance of personal image and breath malodor may be an important factor in social communication [36], there is a constant pressure to look and smell good; halitosis is undesirable [12,38], may create insecurity and interfering with social situations [29,34].

In the literature there are articles concerning general anxiety [28,39], emotional impact [36], the Impact of Oral Conditions on the Quality of Life [39], oral health impact on QoL [34], but no articles were found regarding the impact of halitosis on OoL and the end one may say halitosis affects all aspects of quality of life in any person, specially emotional and social aspects, but with greater impact on obese patients who have halitosis.

### 4.1 Strength and weakness

Strength:

• Reasonable number of cases.
• Prospective case series study.
• Multidisciplinary, bariatric surgeon. Anesthetist, otolaryngologist, maxillofacial, general surgeon were working as team.

Weakness:

• Small number in compatible groups for comparison.
• QOL questionnaire and organoleptic measurement were not classical, but evaluated by 2 different groups of academic physicians.

## 5 Conclusions

The quality of life of overweight and obese, especially emotional and social aspects was significantly disturbed by halitosis more than normal weight subjects with halitosis, while overweight and obesity without halitosis has less impact on the emotional and social aspects of quality of life. Halitosis will affect all aspects of the quality of life of patients overweight, obese, and even average weight patients’ especially emotional and social aspects.

## Provenance and peer review

Not commissioned, externally peer reviewed.

## Ethical approval

Approved by Ethics Committee –College of Medicine- University of Sulaimani No. 2 on 10/10/2018.

## Sources of funding

No any source of funding and no funds was received.

## Author contribution

**Table:** 

Author name	Category 1			Category 2	Category 3
	Conception and design of the study	Acquisition of data (clinical or Laboratory)	Data Analysis and/or interpretation	Drafting of manuscript or critical and/or revision	Approval of final version of the manuscript
Hiwa O. Ahmed	✓		✓	✓	✓
Sherko S. Zmnako	✓			✓	✓
Zanyar M. Amin	✓			✓	✓
Rajan F. Ezzat	✓			✓	✓
Aram Kakarash.	✓			✓	✓
Shahen H. Omer	✓	✓			✓
Hawbash Othman,	✓	✓			✓
Bawan Sherif.	✓	✓			✓

## Conflicts of interest

No any conflict of interest to be declare.

## Registration unique identifying number (UIN)

Research Registry UIN 4430.

## Guarantor

Professor Dr Hiwa Omer Ahmed.
